# Multimodal interventional bronchoscopy for chronic pulmonary *Aspergillus* infection with post-tubercular bronchial occlusion: a case report

**DOI:** 10.3389/fmed.2026.1797581

**Published:** 2026-05-15

**Authors:** Wenjie Wu, Huaxu Yin, Xing Chen, Yanxia He, Bing Xue, Jie Zhang, Ting Wang

**Affiliations:** Department of Respiratory and Critical Care Medicine, Chuiyangliu Hospital Affiliated to Tsinghua University, Beijing, China

**Keywords:** chronic pulmonary aspergillosis, cryotherapy, EBUS-TBNA, interventional bronchoscopy, pulmonary tuberculosis

## Abstract

Chronic pulmonary aspergillosis (CPA) is an increasingly recognized complication of both active and treated pulmonary tuberculosis (PTB). Optimal treatment for CPA in PTB patients has not been established. Here, we report a 36-years-old female with a history of childhood pulmonary tuberculosis presenting with persistent low-grade fever after COVID-19 infection. Imaging showed right upper lobe consolidation and bronchial occlusion. Laboratory investigations revealed positive bronchoalveolar lavage fluid (BALF) and serum galactomannan, and microbiological analyses including targeted Next-Generation Sequencing (tNGS) identified *Aspergillus terreus*, consistent with a diagnosis of CPA. Systemic voriconazole was ineffective due to subtherapeutic drug levels and was complicated by visual adverse effects. Multimodal interventional bronchoscopy was performed, including Endobronchial Ultrasound-guided Transbronchial Needle Aspiration (EBUS-TBNA), electrosurgical incision, balloon dilation, cryotherapy, and local antifungal perfusion. Clinical and radiological improvement was achieved, with resolution of fever, lesion absorption on CT, and negative microbiological and GM results. This case indicates that customized multimodal interventional bronchoscopy is a feasible and effective strategy for post-tubercular chronic pulmonary aspergillosis with bronchial occlusion when systemic antifungals are not optimal.

## Introduction

The incidence and diagnosis of chronic pulmonary aspergillosis (CPA) have shown a clear upward trend worldwide in recent years (1). This rise is driven by heightened clinical awareness, advances in diagnostic modalities, and an expanding population of at-risk individuals. The common underlying diseases in CPA patients include pulmonary tuberculosis, non-tuberculous mycobacterial infections, allergic bronchopulmonary aspergillosis (ABPA), chronic obstructive pulmonary disease (COPD), bronchiectasis, pulmonary fibrosis with lung cysts, pneumoconiosis, pulmonary cystic disease, sarcoidosis, history of pneumothorax, and post-lung cancer surgery, among others (2, 3).

Pulmonary tuberculosis stands out as the most important underlying disease (4). Globally, the estimated prevalence of CPA is 3 million cases, among which 1.2 million are attributable to complications of pulmonary tuberculosis (5). However, this proportion varies substantially across regions. In countries with low tuberculosis prevalence, non-tuberculous mycobacterial infections and COPD become crucial (6). Studies have demonstrated that 21% (United States of America) to 35% (Taiwan, China) of PTB patients develop pulmonary cavities, with approximately 22% of these cavity-forming patients subsequently progressing to CPA (5, 7). These patients present with destructive and fibrotic changes in the lung parenchyma, which result in airway narrowing and obstruction (8). Such changes may induce airflow stasis and *Aspergillus* colonization or proliferation in bronchioles (9, 10).

The guidelines formulated by the European Society for Clinical Microbiology and Infectious Diseases (ESCMID), the European Confederation of Medical Mycology (ECMM), and the European Respiratory Society (ERS) define five subtypes of CPA, including simple aspergilloma (SA), chronic cavitary pulmonary aspergillosis (CCPA), chronic fibrosing pulmonary aspergillosis (CFPA), subacute invasive aspergillosis (SAIA), as well as aspergillus nodule (AN) (11). Surgical resection of aspergilloma represents an important therapeutic strategy for CPA patients with adequate pulmonary function, and is particularly the preferred approach for SA. However, many patients are physically frail, which confers an increased risk of mortality and perioperative complications. Antifungal therapy is therefore considered for patients ineligible for surgery. Triazoles (itraconazole, voriconazole, posaconazole, and isavuconazole) constitute the only drug class available for oral treatment of CPA and serve as first-line therapy for CCPA and SAIA (7, 12). Nonetheless, triazoles are associated with frequent adverse effects, including peripheral neuropathy, heart failure, elevated liver enzymes, QTc prolongation, and photosensitivity (13).

Multimodal bronchoscopic intervention enables precise lesion debridement and improved airway drainage with minimal trauma and high safety, effectively compensating for the limitations of surgery and medication, offering an innovative strategy for post-tuberculosis chronic pulmonary aspergillosis. Here, we report an unusual case of CPA with post-tubercular bronchial occlusion in an immunocompetent young female patient, who achieved a favorable clinical outcome following multimodal interventional bronchoscopy combined with systemic antifungal therapy. This case indicates that customized multimodal interventional bronchoscopy is a feasible and effective strategy for post-tubercular chronic pulmonary aspergillosis with bronchial occlusion.

## Case report

A 36-years-old female with persistent low-grade fever was referred to our hospital. She had been treated for pulmonary tuberculosis for approximately 2 years at the age of 11. Three years ago (December 2022), she developed low-grade fever (37.0 °C–37.4 °C) following a COVID-19 infection. Chest CT demonstrated right upper lobe consolidation, and bronchoscopy identified anterior segment bronchial stenosis with yellowish-white purulent secretions in the distal bronchi. External hospital records showed that fungal culture of the secretion indicated *Aspergillus* spp., and serum and BALF galactomannan (GM) test were positive, with no quantitative data available. She was treated with oral voriconazole 200 mg, q12h.

Nine months later (September 2023), follow-up chest CT showed no improvement in the right upper lobe lesions. Repeat bronchoscopy revealed cicatricial occlusion of the right upper lobe anterior segment bronchus. Endobronchial ultrasound-guided transbronchial needle aspiration (EBUS-TBNA) was performed, aspirating purulent secretions; fungal culture of the aspirate identified *Aspergillus* spp. Therapeutic drug monitoring was performed, with a voriconazole trough concentration of 0.33 μg/mL (recommended therapeutic window: 1.0–5.5 μg/mL), suggestive of a subtherapeutic level. The voriconazole dosage was therefore increased to 300 mg every 12 h. However, 3 weeks later, the patient developed visual disturbances. Voriconazole-induced adverse effects were suspected, and the drug was promptly discontinued. Surgical intervention was recommended but refused by the patient.

**FIGURE 1:**
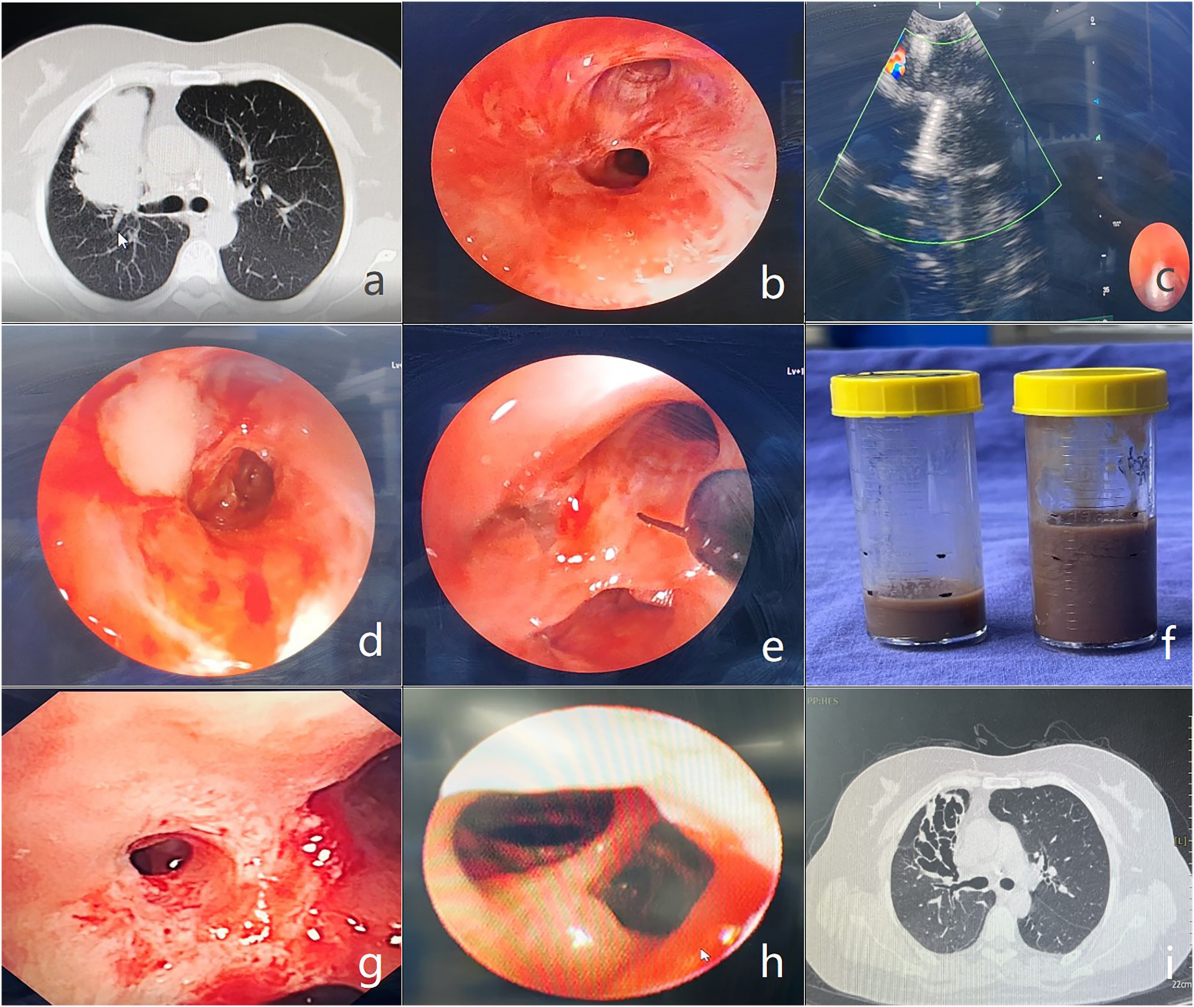
**(a)** Chest CT revealed consolidation in the right upper lobe; **(b)** bronchoscopy revealed complete occlusion of the right upper lobe anterior segment bronchus; **(c)** endobronchial ultrasound-guided transbronchial needle aspiration (EBUS-TBNA) was performed with meticulous avoidance of perilesional blood vessels; **(d)** purulent secretion was noted oozing from the puncture site; **(e)** the overlying scar tissue at the puncture site was incised using an electrical knife; **(f)** a large volume of brown, viscous secretion was drained; **(g)** the bronchial incision was further enlarged; **(h)** distal bronchiectasis was noted via an ultra-thin bronchoscope; **(i)** follow-up chest CT revealed the disappearance of consolidation in the right upper lobe.

Since then, the patient continued to experience intermittent low-grade fever (37.0 °C–37.4 °C). She presented to our outpatient clinic and was subsequently admitted on October 16, 2025. Chest CT revealed persistent right upper lobe lesions (Figure 1a), with several new lesions identified in the right upper lobe apical segment. Bronchoscopy was performed, which demonstrated complete occlusion of the right upper lobe anterior segment bronchus (Figure 1b).

Under EBUS guidance, we performed EBUS-TBNA with meticulous avoidance of perilesional blood vessels (Figure 1c). A small amount of yellowish-white purulent secretion was aspirated into the negative-pressure syringe. Upon withdrawal of the puncture needle, purulent secretion was noted oozing from the puncture site (Figure 1d). Subsequently, we carefully incised the overlying scar at the puncture point with an electrosurgical knife (Figure 1e), from which a large volume of brown, viscous secretion was drained (Figure 1f). The BALF GM test result was 5.433 (CPA cutoff > 1.5) and targeted next-generation sequencing (tNGS) of the secretions identified *Aspergillus terreus*, with a sequence read count of 2,970.

Three days later, repeat bronchoscopy was performed. The bronchial incision was further enlarged using an electrosurgical knife, followed by balloon dilation at the incision site, which exposed a distinct bronchial orifice (Figure 1g). An ultra-thin bronchoscope (MP290, Olympus, Japan) was successfully advanced and distal bronchiectasis was noted (Figure 1h). Local freeze-thaw therapy was performed at the bronchial orifice with a 1.1-mm cryoprobe; then, voriconazole solution (20 mg voriconazole in 20 mL normal saline) was delivered via topical perfusion to the distal bronchial segments. Given the presence of a few new lesions in the apical segment of the right upper lobe bronchus, as well as previous adverse effects (visual disturbances) and poor efficacy with oral voriconazole, the patient was prescribed oral isavuconazole capsules at 200 mg once daily (double dose for the first 2 days).

The patient recovered well after bronchoscopy and has not experienced any further low-grade fever. Outpatient follow-up was given 1 month later (November 2022). Follow-up chest CT revealed the disappearance of consolidation in the right upper lobe, with visible bronchiectasis (Figure 1i), and the new lesions in the apical segment of the right upper lobe bronchus had been absorbed. Bronchoscopy demonstrated good drainage in the anterior segment of the right upper lobe, and no further purulent secretions were observed in the bronchial lumen. The GM tests of BALF and serum were negative. Oral isavuconazole was discontinued, and the patient was advised to continue follow-up to prevent recurrence. At the last follow-up (March 31, 2026), chest CT revealed no lesion recurrence.

## Discussion

The *Aspergillus* genus comprises more than 400 distinct species (14). Epidemiological evidence consistently identifies *Aspergillus fumigatus* as the predominant species implicated in chronic pulmonary aspergillosis (CPA) (13). Notably, a prospective international multicenter surveillance study demonstrated that *Aspergillus terreus* was the most frequently isolated taxa from patients with chronic lung diseases, with an isolation rate of 39.2% (15). Nevertheless, it remains to be fully elucidated whether *Aspergillus terreus* represents an emerging pathogen within this disease entity.

Triazoles represent the only oral drug class for the treatment of CPA and constitute the first-line therapy for CCPA and SAIA (12). Four triazole agents–itraconazole, voriconazole, posaconazole, and isavuconazole–are currently approved for CPA management. Itraconazole and voriconazole exhibit comparable efficacy (16, 17); however, itraconazole is associated with fewer adverse effects, rendering it the preferred initial treatment (4). In select cases, inhaled (nebulized) amphotericin B may help sustain clinical improvement, particularly in patients with extensive disease refractory to oral azole therapy. Notably, *Aspergillus terreus* responds poorly to amphotericin B and is thus considered resistant to this agent.

Unlike invasive aspergillosis (IA), which predominantly occurs in immunocompromised patients, chronic pulmonary aspergillosis (CPA) affects apparently immunocompetent individuals, usually with a pre-existing lung condition, although it may have been clinically silent (13). Optimal treatment strategies for CPA complicating PTB lung disease remain poorly defined and have not yet been formally established in clinical practice, largely due to the complex characteristics of PTB-associated lung damage and the unique clinical challenges posed by the coexistence of these two conditions. PTB patients often present with irreversible structural lung lesions such as bronchial stenosis, cavitation, pulmonary fibrosis, or pleural adhesions, which not only create a hypoxic and nutrient-rich microenvironment conducive to *Aspergillus* colonization and invasive growth but also compromise the local drug delivery and tissue penetration of systemic medications (4, 18). Consequently, systemic antifungal therapy, the conventional first-line approach for CPA, is far from universally effective in this specific patient population; even with prolonged courses of standard antifungal agents, clinical and radiological remission rates remain suboptimal, and drug-related adverse effects are frequently reported. These adverse events–including hepatotoxicity, nephrotoxicity, hematological abnormalities, and the visual impairment observed in the present patient–frequently lead to treatment discontinuation or dose reduction and ultimately limit therapeutic efficacy (11).

For PTB patients with CPA who have favorable pulmonary reserve and localized, resectable lesions (e.g., solitary cavitary lesions with no extensive bilateral involvement), surgical resection has been proposed as a potential alternative treatment strategy to eradicate the *Aspergillus*-infected foci. However, this surgical approach ranks among the most technically challenging thoracic procedures, owing to the severe intra-thoracic adhesions, distorted bronchopulmonary anatomy, and impaired pulmonary function inherent to PTB-related lung disease. As a result, surgical resection for PTB-associated CPA is associated with considerable perioperative mortality and morbidity, including severe bleeding, bronchopleural fistula, acute respiratory failure, and recurrent infection, which severely restricts its clinical applicability and limits it to a highly select subset of patients (19, 20).

Given the limitations of systemic antifungal therapy and surgery, local antifungal instillation represents a promising adjunctive or alternative strategy for pulmonary aspergillosis, with the primary advantage of delivering high concentrations of antifungal drugs directly to the infected foci while minimizing systemic exposure and adverse effects. In this case, following bronchial recanalization, a topical antifungal agent was perfused into the distal bronchiectatic lumen, enabling targeted delivery to *Aspergillus* colonization sites, with encouraging outcomes. Various antifungal agents–including amphotericin B, voriconazole, miconazole, ketoconazole, and fluconazole–have been used for local instillation (21–25). In the present case, based on the etiological identification of *Aspergillus terreus*, voriconazole was selected for local perfusion because this isolate is resistant to amphotericin B and fluconazole exhibits no activity against *Aspergillus* species.

In this case, we utilized a combination of multiple interventional bronchoscopic modalities, including EBUS-TBNA, electrosurgical incision, balloon dilation, cryotherapy, and subsequent local antifungal instillation. We hypothesize that the synergistic effect of these techniques overcomes the inherent limitations of both single-modality local therapy and systemic antifungal treatment: EBUS-TBNA allows accurate sampling and histopathological diagnosis of infected lesions, while enabling precise localization of occluded bronchial orifices; electrosurgical incision and balloon dilation alleviate bronchial stenosis and restore airway patency; cryotherapy was employed to prevent local restenosis at the bronchial orifice; and targeted local antifungal instillation delivers high-dose antifungal agents to the debrided and patent lesion site, thereby exerting sustained antifungal activity. This combined interventional approach resulted in significant clinical improvement (resolution of persistent low-grade fever), and radiological regression of CPA lesions without severe adverse events.

## Conclusion

This case illustrates the clinical potential of a customized multimodal bronchoscopic interventional strategy for the management of PTB-associated CPA. Although further large-scale prospective studies are required to confirm the safety, efficacy, and long-term outcomes of this combined interventional modality, our experience indicates that it may serve as a novel, feasible, and valuable therapeutic option for selected patients with PTB-associated CPA who have failed or are intolerant to systemic antifungal therapy and are ineligible for surgical resection. In addition, this case underscores the importance of a multidisciplinary team (including interventional pulmonologists, infectious disease specialists, and thoracic surgeons) in the individualized management of this complex and difficult-to-treat patient population.

## Data Availability

The original contributions presented in this study are included in this article/supplementary material, further inquiries can be directed to the corresponding author.
